# Bio-recognitive photonics of a DNA-guided organic semiconductor

**DOI:** 10.1038/ncomms10234

**Published:** 2016-01-04

**Authors:** Seung Hyuk Back, Jin Hyuk Park, Chunzhi Cui, Dong June Ahn

**Affiliations:** 1 KU-KIST Graduate School of Converging Science and Technology, Korea University, Seoul 02841, Korea; 2 Department of Chemical and Biological Engineering, Korea University, Seoul 02841, Korea; 3 Center for Theragnosis, Biomedical Research Institute, Korea Institute of Science and Technology, Seoul 02792, Korea

## Abstract

Incorporation of duplex DNA with higher molecular weights has attracted attention for a new opportunity towards a better organic light-emitting diode (OLED) capability. However, biological recognition by OLED materials is yet to be addressed. In this study, specific oligomeric DNA–DNA recognition is successfully achieved by tri (8-hydroxyquinoline) aluminium (Alq_3_), an organic semiconductor. Alq_3_ rods crystallized with guidance from single-strand DNA molecules show, strikingly, a unique distribution of the DNA molecules with a shape of an ‘inverted' hourglass. The crystal's luminescent intensity is enhanced by 1.6-fold upon recognition of the perfect-matched target DNA sequence, but not in the case of a single-base mismatched one. The DNA–DNA recognition forming double-helix structure is identified to occur only in the rod's outer periphery. This study opens up new opportunities of Alq_3_, one of the most widely used OLED materials, enabling biological recognition.

## Abstract (web summary)

BioLEDs is an emerging group of light-emitting diodes that use duplex-strand DNA to enhance luminescence intensity. Here, Back *et al.* show that only the specific binding between a pair of single-strand DNA can trigger the enhancement, which potentially makes BioLEDs an easy platform for DNA recognition.

## Graphical abstract

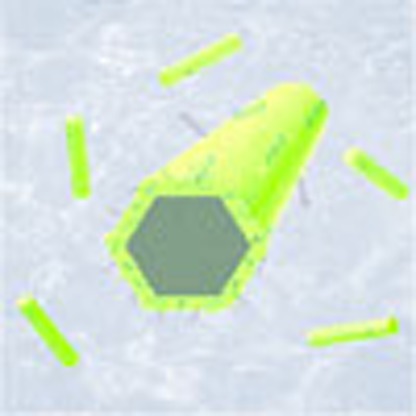

Novel display materials have gained keen attraction recently in the fields of electronics and photonics research especially owing to the rapid evolution of smart communication devices^1^,^2^,^3^. Among the various display materials available, organic semiconductors or metal-organic compounds are considered to be very promising, and they have therefore been intensely investigated^4^,^5^,^6^. An alumina quinoline, tri (8-hydroxyquinoline) aluminium (Alq_3_), first reported approximately three decades ago, which emits in the green and blue spectra, is a material of central interest^7^,^8^,^9^. Alq_3_ is currently used in a multitude of organic light-emitting diodes (OLEDs)^10^,^11^,^12^,^13^ that are used in various displays. Since it was first reported, enormous improvements have been made in its light emission efficiency, to provide higher display quality^14^,^15^,^16^,^17^,^18^. One peculiar approach incorporates a biological material into the light-emitting device, often called a BioLED^19^. An example is DNA in the form of a thin film introduced within a conventional electroluminescent cell incorporating an Alq_3_ layer^20^. Utilized in the device were double-strand DNAs (dsDNAs) extracted from natural organisms and complexed with cationic surfactants; the device provides ∼30-fold increase in luminescence intensity^21^. This phenomenon was attributed to the contribution of the DNA layer to the electron blocking effect, thus reducing significant loss of electrons and enhancing electron–hole recombination in the cell^20^. Luminescent dyes entrapped within dsDNA thin films reported^22^ also exhibited higher intensity owing to less non-radiative relaxation. This novel capability of DNA is noteworthy as a gadget in light-emitting devices. The value of the devices can be recognized even higher as they incorporate the ‘bio-recognition function'. Current BioLEDs now face a new journey to the realm of biological recognition.

To this end, this study presents a critical step endowing an OLED material with a biological recognition function. We demonstrate for the first time that only specific DNA–DNA recognition triggers photoluminescent enhancement reflected by Alq_3_, the most widely used OLED material.

## Results

### Optical properties analyses of DNA-guided Alq_3_ rods

**Figure 1:**
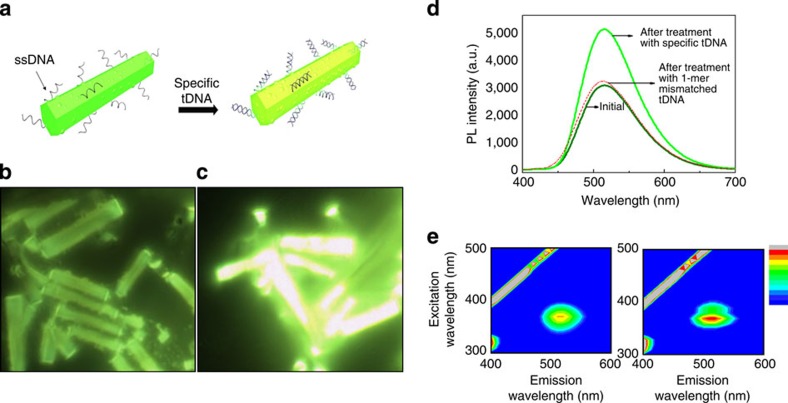
Optical effects of DNA-guided Alq_3_ rods. (**a**) Schematic illustration of the recognition of specific tDNA by Alq_3_ rod crystals. (**b**,**c**) Colour CCD images of the samples before and after interaction with tDNA, respectively. (**d**) PL spectra of the initial Alq_3_ crystals (indicated by dark green line), and after interaction with specific tDNA (green line) and 1-mer mismatched DNA (red dotted line), respectively, with excitation at 365 nm. (**e**) PLE spectra of the samples before and after interaction with tDNA, respectively.

We first observed the characteristic alteration when Alq_3_ particles incorporating single-strand DNA (ssDNA) moieties interacted with specific target DNA (tDNA) molecules. Crystallization of Alq_3_ has been conventionally executed with the aid of surfactants and recently become successful using ssDNA molecules only^23^. With guidance from ssDNA, we fabricated prismatic hexagonal rod crystals composed of Alq_3_. In this study, the oligomeric ssDNA used for crystal guidance was a 27-mer sequence of anthrax lethal factor. Figure 1a shows a schematic illustration of the recognition of specific tDNA by the light-emitting Alq_3_ rod crystallized by ssDNA. Figure 1b,c provide colour charge-coupled device (CCD) images of the ssDNA-guided Alq_3_ (ssDNA-Alq_3_) rods before and after treatment with tDNA molecules, respectively. We observed the ssDNA-Alq_3_ rods emitting green luminescence. Interestingly, the intensity of the green luminescence of the ssDNA-Alq_3_ rods was markedly enhanced after interaction with specific tDNA molecules. For quantitative analysis of the intensity enhancement, we measured the photoluminescence (PL) spectra of the Alq_3_ rods. As shown in Fig. 1d, a broad PL peak was observed at ∼512 nm when samples were excited with a laser at 365 nm, which corresponds to the main absorption band of Alq_3_. The PL spectra were yellowish-green, composed of both α and δ phases^8^,^9^,^24^. After interaction with specific tDNA molecules, the PL peak intensity increased ∼1.6-fold, which is concordant with the results of the CCD analysis. Interestingly, when treated with single-base (1-mer) mismatched tDNA molecules that are less specific, the Alq_3_ rods showed little enhancement of PL intensity. In addition, PL excitation (PLE) spectrum analysis confirmed the enhancement of PL intensity, as shown in Fig. 1e. The intensity with excitation at 365 nm and emission at 512 nm was clearly higher following treatment with specific target molecules.

### Crystal structure analyses upon interaction with DNA

**Figure 2:**
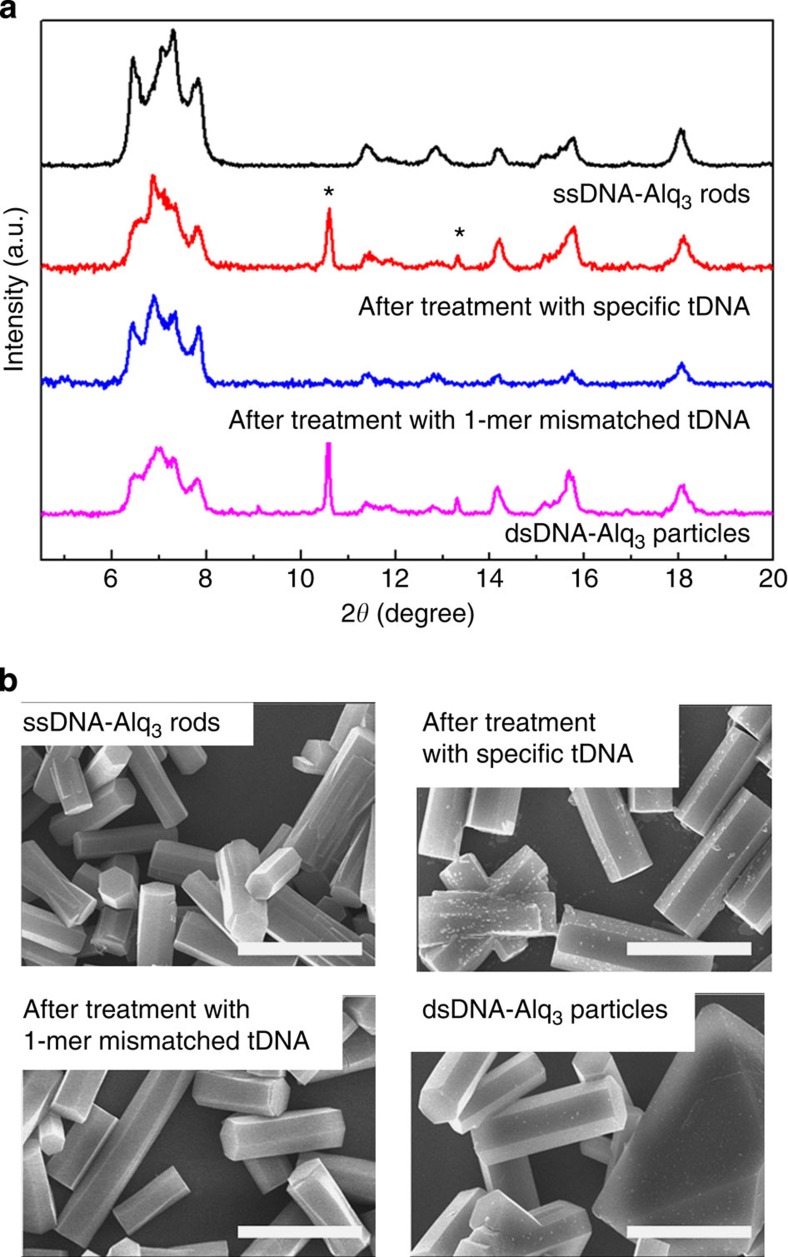
Crystal structure and morphology of Alq_3_ rods upon interaction with DNA. (**a**) XRD patterns and (**b**) SEM images of ssDNA-Alq_3_ rods, ssDNA-Alq_3_ rods treated with specific tDNA, ssDNA-Alq_3_ rods treated with 1-mer mismatched tDNA and dsDNA-Alq_3_ particles in which dsDNA formed *a priori* (scale bars, 10 μm).

To further explore the PL enhancement of the Alq_3_ rods after interaction with specific tDNA molecules, we selected four crystal samples of ssDNA-Alq_3_, ssDNA-Alq_3_ treated with specific tDNA, ssDNA-Alq_3_ treated with 1-mer mismatched tDNA and dsDNA-Alq_3_ (dsDNA-guided Alq_3_ rods crystallized by the use of dsDNA molecules from the start). X-ray diffraction (XRD) patterns were observed, as shown in Fig. 2a, to examine structural changes in the Alq_3_ crystals. The XRD pattern of the ssDNA-Alq_3_ rod showed typical α-phase peaks for Alq_3_ at 11.40° and 12.81°, along with a δ-phase peak at 11.79°. Hence, the ssDNA-Alq_3_ rods fabricated in this study contain both α- and δ-phases^8^,^9^,^25^,^26^,^27^, which is consistent with the yellowish-green luminescence observed in the PL analyses. Upon interaction with specific tDNA molecules, the rods showed an XRD pattern almost identical to that of the initial Alq_3_ rods, except that two additional peaks appeared at 10.59° and 13.31°. The source of these two peaks was identified as the dsDNA forming the helical structure^28^,^29^. These two peaks were also evident in the dsDNA-Alq_3_ sample, in which the double-helix DNA structure was formed before the crystallization. In a very clear comparison, these two peaks were completely absent in the case of ssDNA-Alq_3_ rods treated with 1-mer mismatched tDNA molecules, indicating that nearly no helical dsDNA was present. The morphological features of the four Alq_3_ crystals were observed by field-emission scanning electron microscopy (SEM), as shown in Fig. 2b. The ssDNA-Alq_3_ rods showed a regular prismatic hexagonal shape with a smooth surface, similar to the morphology of Alq_3_ rods fabricated using the surfactant, cetyltrimethylammonium bromide (Supplementary Fig. 1)^30^. Upon interaction with specific tDNA, the initially smooth surface of the Alq_3_ rods became rough. A rough surface was not observed either following treatment with 1-mer mismatched tDNA or in the sample of dsDNA-Alq_3_ rods. Therefore, the Alq_3_ rods only showed a significant enhancement in PL following interaction with specific tDNA, accompanied by the following interesting features: diffraction peaks indicative of double-helix DNA and surface roughening.

### Layers in a single Alq_3_ rod and DNA distribution

**Figure 3:**
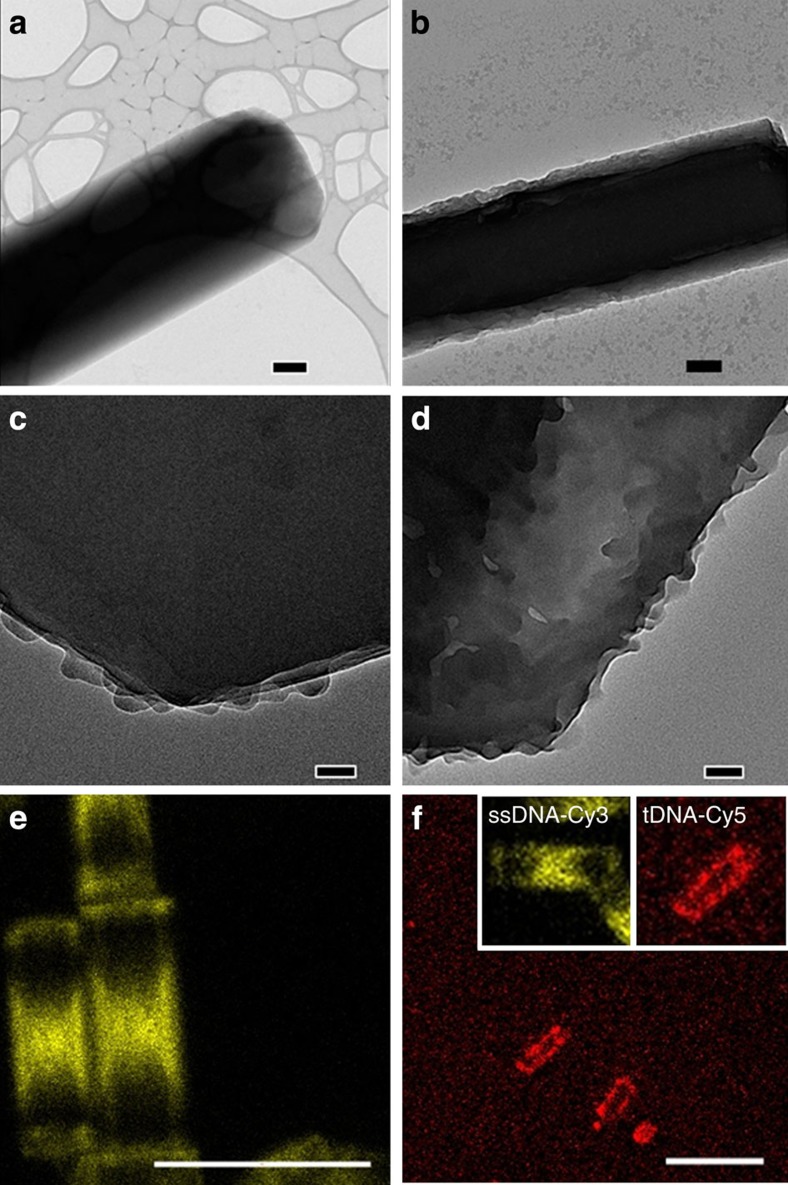
Observation of inner core and crust layers in Alq3 rod with distributions of DNA molecules. HR-TEM images of a ssDNA-Alq_3_ rod (**a**,**c**) before and (**b**,**d**) after being treated with the specific tDNA: (**a**,**b**) top view (scale bar, 200 nm) and (**c**,**d**) near edge (scale bar, 50 nm). CLSM images captured at the centre plane with excitation by a 555-nm laser of (**e**) the ssDNA-Cy3 molecules used for crystal guidance (scale bar, 20 μm) and (**f**) the perfect-match tDNA-Cy5 molecules (scale bar, 20 μm) recognized by the surrounding peripheral ssDNA. (Insets represent respectively the ssDNA-Cy3 and tDNA-Cy5 distributions after hybridization.) The filters used for observing the Cy3 and Cy5 dyes ranged from 300 to 630 nm and 630 to 800 nm, respectively.

To observe the roughened surface more closely, we employed high-resolution transmission electron microscopy (HR-TEM) of the ssDNA-Alq_3_ rods, before and after interaction with specific tDNA molecules. In comparison with the prismatic hexagonal shape of the ssDNA-Alq_3_ rods (Fig. 3a), interaction with specific tDNA resulted in formation of an 120-nm thick crust layer surrounding the inner core of the rod of which the whole thickness is 800 nm, as shown in Fig. 3b. In the magnified HR-TEM images of regions near the rod edges as shown in Fig. 3c,d, the surface crust layer was observable to a small degree in ssDNA-Alq_3_ rod, but increased markedly upon specific tDNA interaction. We can estimate, from the HR-TEM image (Fig. 3b), the volume ratio of the crust layer in the Alq_3_ rod to be 50 vol% (Supplementary Fig. 2). It is noted that such a crust layer was completely absent in the reference case, the cetyltrimethylammonium bromide-guided Alq_3_ rod (Supplementary Fig. 1). Hence, it is rational that the increased crust layer was induced by the recognition of specific tDNA by the ssDNA present in the Alq_3_ rod. To confirm the positioning of the DNA molecules, we used ssDNA and specific tDNA molecules labelled with Cy3 and Cy5 fluorescent dyes, respectively. The distribution of the DNA molecules was visualized by tracing the corresponding dye moieties using a confocal laser scanning microscope (CLSM) capable of excitation with a 555-nm laser with a variable-wavelength filter. Strikingly to observe in Fig. 3e, the ssDNA-Cy3 molecules showed a very unique distribution in the shape of an ‘inverted' hourglass over the Alq_3_ rod. DNA molecules have recently been shown to play a role in crystallization^23^ and to act as an alternative to surfactants. A conventional concept of crystallization regarding the role of surfactant molecules (that is, wrapping around particulate seeds and resulting in subsequent crystallization) was found to be invalid for the DNA molecules used for the crystal guidance, at least in the present case. A detailed understanding of why the ssDNA molecules are distributed in this unique manner is presently lacking. How the ssDNA molecules having the inverted hourglass distribution interact with the tDNA molecules is investigated next. Tracing of the dye moieties showed that the tDNA-Cy5 molecules were only present on the rod's outer periphery, which emitted red fluorescence as shown in Fig. 3f. Interestingly, the unique distribution of ssDNA molecules is nearly maintained. Thus, we can infer that recognition of the tDNA molecules by the ssDNA molecules occurred only in the region limited to the roughened surface but not in the inner core. Hence, the abovementioned 120-nm thick surface crust, shown in Fig. 3b, was induced by specific DNA–DNA recognition.

### Depth-wise identification of localized photoluminescence

**Figure 4:**
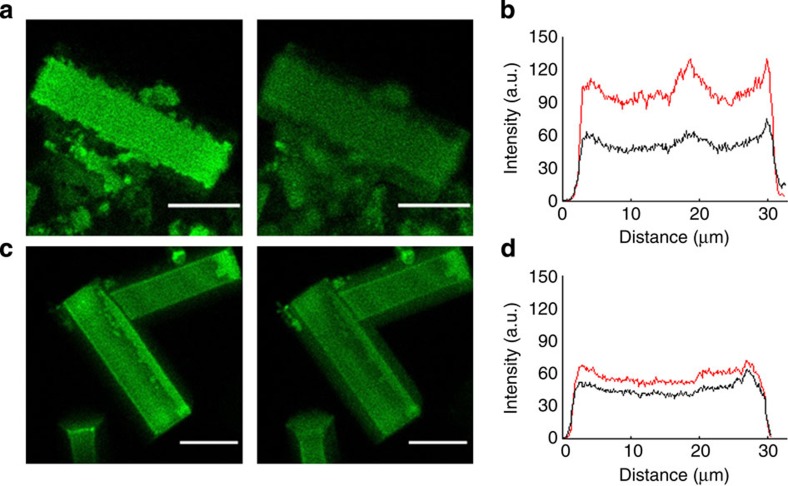
Depth-wise identification of localized PL of Alq_3_ molecules. (**a**) CLSM images of the upper plane (left) and the 2.5-μm inner plane (right) of a tDNA-recognized ssDNA-Alq_3_ rod, (**b**) longitudinal PL profiles in which the intensity of the upper plane (indicated by red line) is ∼2-fold higher than that of the inner plane (black line). (**c**) Images and (**d**) PL profiles of ssDNA-Alq_3_ rods are also shown. The images and profiles were obtained with excitation by a 405-nm laser; the filter used for observation of Alq_3_ molecules ranged from 300 to 550 nm (scale bars, 10 μm).

In the next stage, we investigated precisely where in the Alq_3_ rods the enhanced PL originates, that is, the inner core, the surface crust, or the entire rod. To compare the PL intensity of the crust layer and inner core of a single Alq_3_ rod, we again employed CLSM, but with excitation at 405 nm, to excite the Alq_3_ molecules. From the rod having thickness of 11 μm, we acquired two-dimensional cutting-plane images, one from the uppermost surface layer and the other at the depth of the 2.5-μm below the surface, and thus below the crust. Specific DNA–DNA recognition occurred and Fig. 4a shows a comparison of the uppermost surface layer (left) and the 2.5-μm inner planes (right), corresponding to the crust layer and the inner core, respectively. We observed that the upper plane was markedly brighter than the inner plane. We quantified this phenomenon using a profiling analysis of the rod crystals in a longitudinal direction, as shown in Fig. 4b; the PL intensity of the upper plane (indicated by red line) was ∼2-fold higher than that of the inner plane (black line). In contrast, the upper plane was a little brighter than the lower in the ssDNA-Alq_3_ rod, as shown in Fig. 4c,d. Therefore, careful comparison of the PL intensity of the crust layer and the inner core, in addition to analyses of structural variation and molecular profiling, enables us to conclude that specific DNA–DNA recognition caused the increase in the surface crust layer, the very region responsible for the PL enhancement. Thus, Alq_3_, one of the most widely used OLED materials, becomes capable of DNA–DNA recognition.

**Figure 5:**
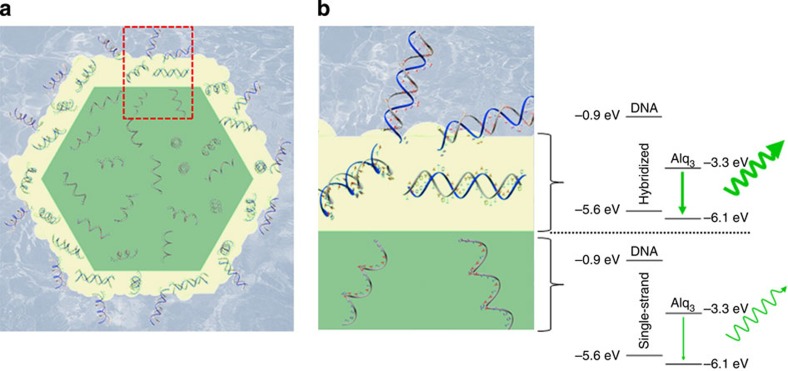
Cross-sectional schematic illustration and energy-level diagram. (**a**) Cross-section of a ssDNA-guided Alq_3_ rod recognizing a specific tDNA at the crust layer and (**b**) enlarged picture indicating double-helix DNA that exerts less non-radiative dissipation.

This novel phenomenon can be explained by the following mechanism: Fig. 5 suggests a cross-sectional schematic illustration of the Alq_3_ rod recognizing the specific tDNA and the energy-level diagrams for both crust layer and inner core. No shift at the main absorption peak of the Alq_3_ rods before and after the recognition was found, which was also true for ssDNA and hybridized rods (Supplementary Fig. 3). This indicates that specific DNA–DNA recognition did not induce any changes in the bandgap of the Alq_3_ rods. As illustrated in the energy diagram, DNA has a wide bandgap such that the lowest unoccupied molecular orbital energy level was −0.9 eV and the highest occupied molecular orbital level was −5.6 eV, whereas the lowest unoccupied molecular orbital and highest occupied molecular orbital levels of the yellowish-green Alq_3_ were −3.3 and −6.1 eV, respectively^20^,^21^. We analysed fluorescence lifetimes to evaluate the effect of the DNA–DNA interaction on luminescence enhancement. The average lifetime was measured to be 1.28 ns for the ssDNA-Alq_3_ rods and evidently increased to 1.63 ns after specific DNA–DNA interaction, indicating that DNA recognition evokes enhanced prevention of non-radiative relaxation of Alq_3_ molecules (Supplementary Fig. 4 and Supplementary Table 1). Hence, the PL intensity is enhanced only in the limited region of the surface crust layer, where specific recognition event occurs.

## Discussion

In summary, the Alq_3_, a molecular organic semiconductor, crystallized and functionalized with ssDNA molecules is found capable of recognizing biological interaction, for the first time. The crystal's luminescent intensity is enhanced by 1.6-fold upon label-free recognition of perfect-matched tDNA sequence, but not in the case of single-base mismatched one. Specific DNA–DNA interaction induces double-helix DNA structure on the crystal's surface crust layer that is analysed to be responsible for longer fluorescence lifetime and the increase in the luminescent response. This study signifies a new direction of OLED materials toward unprecedented bio-recognitive photonic functions and applications.

## Methods

### Fabrication of DNA-guided Alq_3_ rods

Commercial Alq_3_ powder was dissolved in tetrahydrofuran at a concentration of 1 mg ml^−1^, to form a stock solution. The stock solution (2 ml) was injected into 20 ml of various aqueous DNA solutions at a concentration of 0.5 μM, with vigorous stirring (∼800 r.p.m.) for 10 min. The mixture was stored at room temperature (RT) overnight to allow formation of visible precipitate. The ssDNA used in this fabrication had a sequence of NH_2_-5′-ATC CTT ATC AAT ATT TAA CAA TAA TCC-3′; this ssDNA, hybridized with its complementary sequence, was used as the dsDNA.

### Hybridization of DNA molecules

The ssDNA used in this experiment was the amine-terminated anthrax lethal factor probe DNA sequence (NH_2_-5′-ATC CTT ATC AAT ATT TAA CAA TAA TCC-3′). The fabricated ssDNA-Alq_3_ rods were reacted with complementary tDNA (3′-TAG GAA TAG TTA TAA ATT GTT ATT AGG-5′) at a concentration of 0.5 μM at 52 °C for 30 min and then returned to RT. The 1-mer mismatched tDNA sequence used in this study was 3′-TAG GAA TAG TTA CAA ATT GTT ATT AGG-5′.

### Characterization of the Alq_3_ crystal samples

The surface morphology of the Alq_3_ rods was analysed using a field-emission SEM (Hitachi, S-4300) using an acceleration voltage of 15 kV. Powdered samples of Alq_3_ rods were cast on an ultrathin carbon-coated Cu grid or a holey carbon-coated Cu grid and images were captured using an HR-TEM (Tecnai G2, Fei) with an acceleration voltage of 200 kV. The powder XRD (Bruker, D8 Advance with DaVinci) patterns were captured at a voltage of 40 kV, a current of 40 mA and Cu-Kα radiation (*λ*=1.540 Å). The scanning rate was 0.02° s^−1^, and the 2*θ* range was captured from 2° to 20°. A fluorescence spectrophotometer (Hitachi, F-7000) was used for measuring PL and PLE spectra excited by a Xe lamp. A CLSM (Carl Zeiss, LSM700) was used for measuring *z*-sectioning fluorescence images of the isolated single Alq_3_ rod, ssDNA molecules, and tDNA molecules. For this analysis, Alq_3_ rods were fabricated with ssDNA-Cy3 (NH_2_-5′-ATC CTT ATC AAT ATT TAA CAA TAA TCC-3′-Cy3) and tDNA-Cy5 (3′-TAG GAA TAG TTA TAA ATT GTT ATT AGG-5′-Cy5) to visualize the interactions with ssDNA. The Alq_3_ molecules were guided by ssDNA and were excited at 405 nm and detected with a 300–550 nm filter. The ssDNA-Cy3 molecules present in the Alq_3_ rod were excited at 555 nm and detected with a 300–630 nm filter. In addition, the tDNA-Cy5 molecules recognized by the Alq_3_ rods were excited at 555 nm and detected with a 630–800 nm filter. The Alq_3_ rods were analysed using a *z*-stack of images collected at 100 nm intervals through the × 20, × 40 and × 100 objective lenses. Fluorescence lifetimes of the solution-phase samples were obtained with an Edinburgh Instruments FL920 Fluorescence Lifetime spectrometer equipped with 376.6-nm pulsed-diode laser at RT. Quantum yields were measured using IESP-150B (Sumitomo Heavy Industries Advanced Machinery Co. Ltd.) equipped with a Xe lamp (CW500W).

## Additional information

**How to cite this article:** Back, S. H. *et al.* Bio-recognitive photonics of a DNA-guided organic semiconductor. *Nat. Commun.* 7:10234 doi: 10.1038/ncomms10234 (2016).

## Supplementary Material

Supplementary Information Supplementary Figures 1-4 and Supplementary Table 1
