# Supplementary material for: Bio-recognitive photonics of a DNA-guided organic semiconductor
Source: Nat Commun. 2016 Jan 4;7:10234. doi: 10.1038/ncomms10234 (PMC4725759; doi:10.1038/ncomms10234)
Supplement: Supplementary Information — Supplementary Figures 1-4 and Supplementary Table 1 [file ncomms10234-s1.pdf]

Supplementary Figures

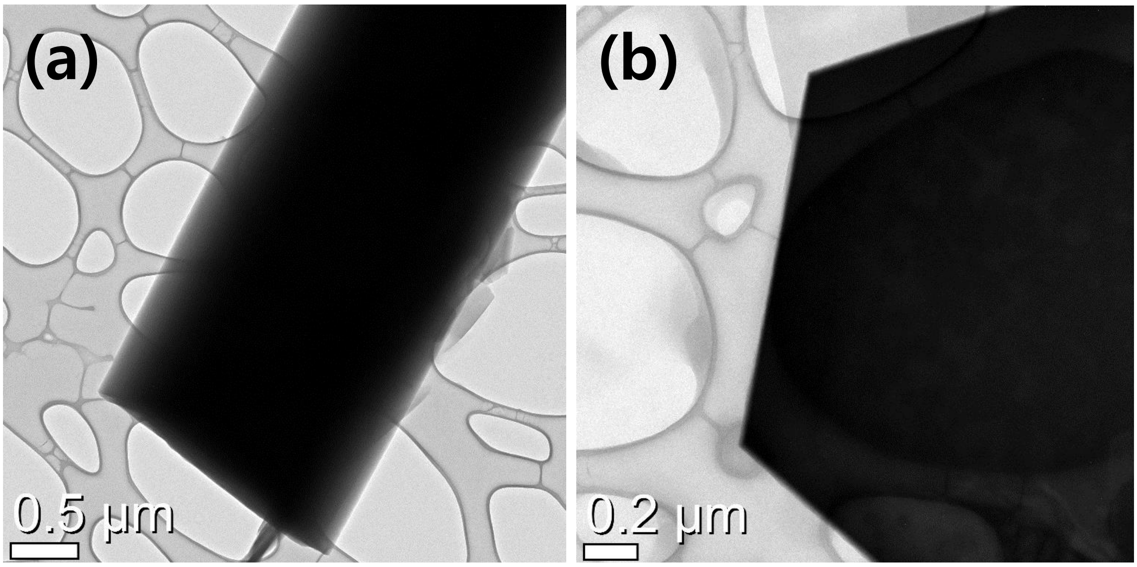

Supplementary Fig. 1 | Morphological analyses of a CTAB-guided Alq_3 rod (a) HR-TEM image and (b) top-view HR-TEM image of the CTAB-guided Alq_3 rod.

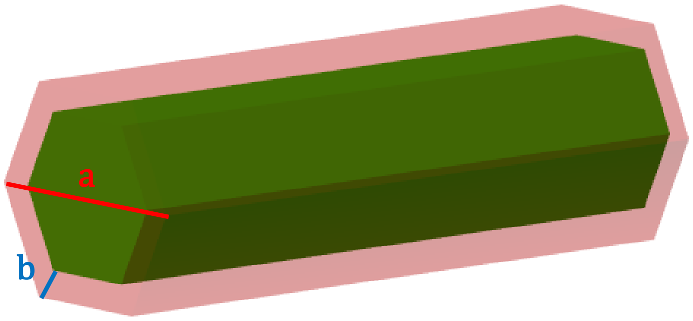

Supplementary Fig. 2 | Volume ratio calculation of the crust layer A 3D schematic illustration of an Alq_3 rod is shown for calculating the volume ratio of the crust layer, where **a** denotes the thickness of the Alq_3 rod and **b** defines the thickness of the crust layer (pink region). The Alq_3 rod was modelled as a regular hexagonal column with the crust layer of uniform thickness. The ratio of **b** to **a**, i.e. the ratio of crust thickness to the rod thickness is estimated to be 16 % from the TEM image. Regarding the volume occupied by the crust layer the following equation is used:

$$R = \frac{V_S}{V} = \frac{4ab - 4b^2}{a^2} \quad (1)$$

Using equation (1), we estimated that the crust layer occupied approximately 50% of the total rod volume.

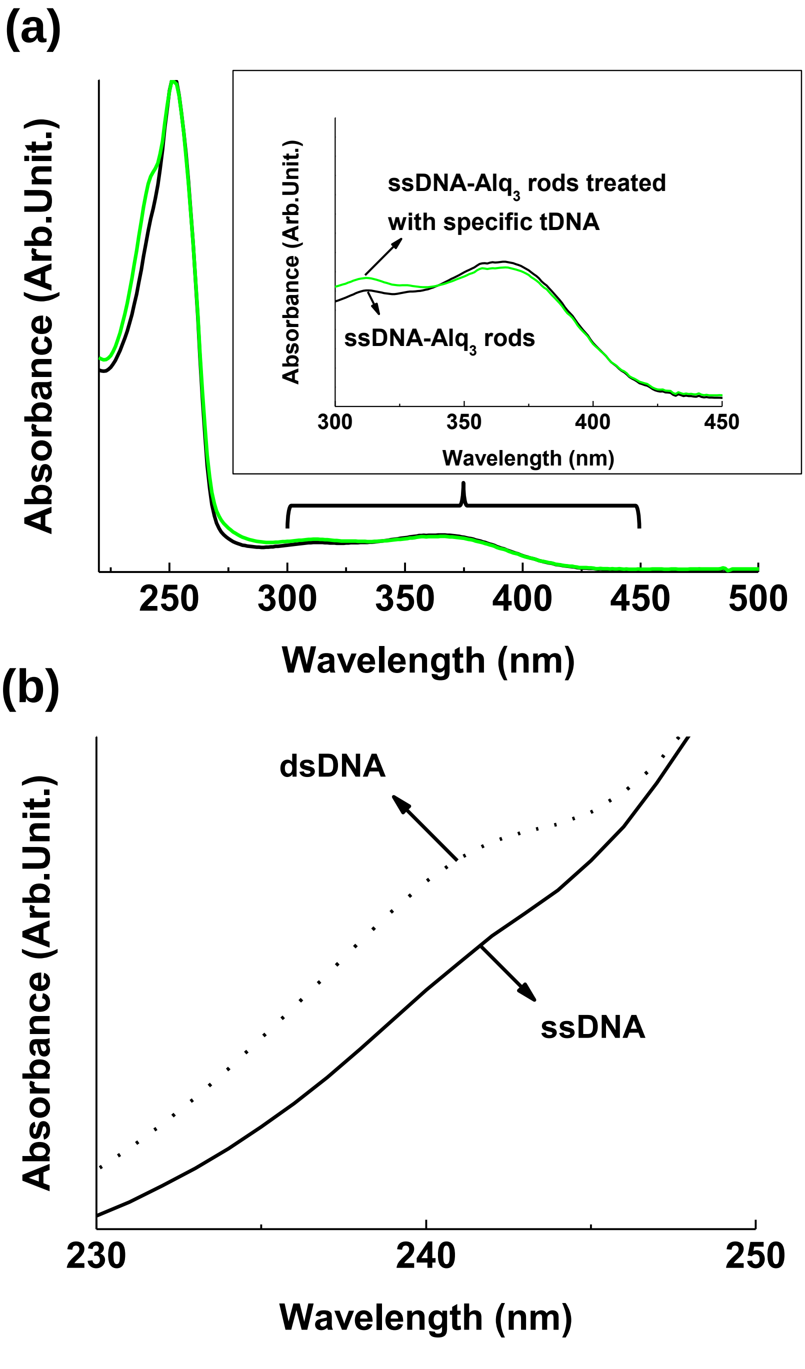

Supplementary Fig. 3 | Absorption spectra analyses (a) Absorption spectra of the ssDNA-Alq_3 rods (black line) and those after treated with specific tDNA (green line). Magnified spectra in the range from 300-450 nm are given inset. (b) Absorption spectra of ssDNA molecules (solid line) and those after hybridized with specific tDNA (dotted line).

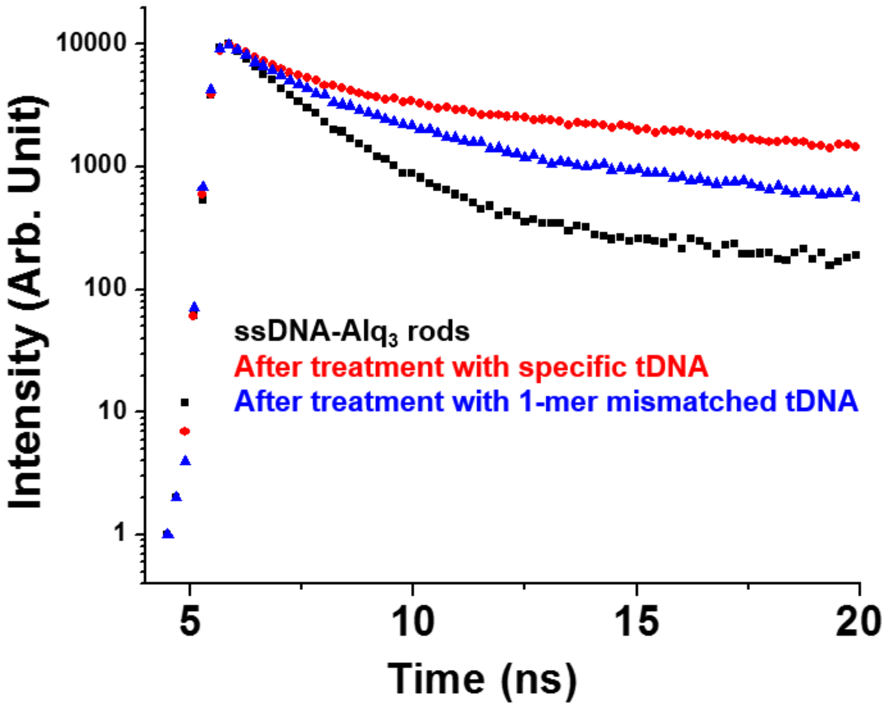

Supplementary Fig. 4 | Fluorescence lifetime observation The fluorescence decay of the initial ssDNA-Alq_3 rods (indicated by dark curve), after treatment with specific tDNA (red curve), and 1-mer mismatched tDNA (blue curve) with excitation at 375 nm.

Supplementary Table

Supplementary Table 1 | Average fluorescence decay and quantum yield of the solution-phase samples

Sample	Average Fluorescence Decay (ns)	Quantum Yield
ssDNA-Alq_3 rods	1.28	0.024
ssDNA-Alq_3 rods treated with specific tDNA	1.63	0.030
ssDNA-Alq_3 rods treated with 1-mer mismatched tDNA	1.40	0.026
